# Spatial-Temporal Speckle Variance in the En-Face View as a Contrast for Optical Coherence Tomography Angiography (OCTA)

**DOI:** 10.3390/s22072447

**Published:** 2022-03-22

**Authors:** Jonathan D. Luisi, Jonathan L. Lin, Bill T. Ameredes, Massoud Motamedi

**Affiliations:** 1 Department of Internal Medicine, University of Texas Medical Branch at Galveston, 301 University Blvd, Galveston, TX 77555, USA; joluisi@utmb.edu (J.D.L.); btamered@utmb.edu (B.T.A.); 2 Department of Ophthalmology and Visual Sciences, University of Texas Medical Branch at Galveston, 301 University Blvd, Galveston, TX 77555, USA; jolin@utmb.edu; 3 Department of Pharmacology and Toxicology, University of Texas Medical Branch at Galveston, 301 University Blvd, Galveston, TX 77555, USA

**Keywords:** optical coherence tomography (OCT), eye, angiography, segmentation, volumetric, low computational power, fast, enhanced contrast, image analysis, generalizable

## Abstract

Optical Coherence Tomography (OCT) is an adaptable depth-resolved imaging modality capable of creating a non-invasive ‘digital biopsy’ of the eye. One of the latest advances in OCT is optical coherence tomography angiography (OCTA), which uses the speckle variance or phase change in the signal to differentiate static tissue from blood flow. Unlike fluorescein angiography (FA), OCTA is contrast free and depth resolved. By combining high-density scan patterns and image processing algorithms, both morphometric and functional data can be extracted into a depth-resolved vascular map of the retina. The algorithm that we explored takes advantage of the temporal-spatial relationship of the speckle variance to improve the contrast of the vessels in the en-face OCT with a single frame. It also does not require the computationally inefficient decorrelation of multiple A-scans to detect vasculature, as used in conventional OCTA analysis. Furthermore, the spatial temporal OCTA (ST-OCTA) methodology tested offers the potential for post hoc analysis to improve the depth-resolved contrast of specific ocular structures, such as blood vessels, with the capability of using only a single frame for efficient screening of large sample volumes, and additional enhancement by processing with choice of frame averaging methods. Applications of this method in pre-clinical studies suggest that the OCTA algorithm and spatial temporal methodology reported here can be employed to investigate microvascularization and blood flow in the retina, and possibly other compartments of the eye.

## 1. Introduction

Optical coherence tomography (OCT), a non-invasive imaging modality that is somewhat analogous to ultrasound, uses reflected, as well as scattered, light to acquire sub-surface images [1]. As light waves are used rather than sound waves, the resolution of OCT is much higher than that of ultrasound, albeit at the cost of shallower penetration (1–2 mm). Its most basic form, the A-scan, is the depth profile of the light wave’s phase change at a single point, where changes in the scattering properties at boundaries in the material slow the reflected beam. However, movement and heterogeneity of the tissue results in excess scattering that presents as “speckle” and, in most applications, is considered noise that requires mitigation. Traditionally, averaging, and other noise reduction methods, are employed with OCT, to reduce the speckle effect on image quality [2,3].

Unlike OCT, in the case of optical coherence tomography angiography (OCTA), the contrast of the speckle pattern is enhanced by the turbidity of moving fluids, i.e., blood flow, which can be advantageous for the detection of movement through blood vessels. The increased scattering of blood in vasculature and presence of large scattering particles, such as blood cells, create transient and localized speckle that can be quantified. Decorrelation algorithms can detect the intensity ‘flicker’ caused by blood flow, especially in capillaries, by rapidly resampling the A-scan phase variance [4,5,6], yielding information about transient speckle or steady flow. For example, the speckle in an optical fundus scope can be mapped to detect flow as previously reported [7,8]. Alternatively, a high-density scan pattern can detect flow, through speckle variance, as increased spatial intensity in the en-face view [4,9,10]. By digitally re-slicing the rectangular B-scan volume to the en-face or C-scan view, the speckle and OCT intensity of the vasculature can be seen to be higher than surrounding tissue [11]. However, since the reflected wave contains both the phase and speckle data, the vasculature is fragmented across the 3D volume and cannot be directly visualized [12,13], which is a shortcoming that requires a solution.

Furthermore, OCTA, as a volumetric imaging modality, has the advantage that it can differentiate between the vascular layers within the inner and outer retina, and, more specifically, localize a vessel in the inner retina to the different vascular plexuses [14]. In a similar theory of enhancing vascular specificity, a comparison of maximum intensity versus mean intensity projection methods showed different specificity in resolving vasculature, allowing for enhancement of microvessels when provided with sufficient scan resolution [15]. Given the potential for additional encoded data in the variance of OCT images, we expanded our methodology to take advantage of decades of advances that have been made in other angiography methods, to develop a novel approach for OCTA analysis, as we describe, below. While retinal vasculature is the established target for which the methods are validated in this paper, this algorithm was also used without major modifications in a corneal neovascularization model [16], demonstrating its utility in different compartments of the eye.

Previously, the spatial-temporal relationship of the speckle variance was developed for fundus angiography for flow measurements; however, the transitive property of this relationship has not been applied to OCTA algorithms. Instead, OCTA algorithms have calculated the variance by either solely a spatial or a temporal approach, with the majority of methods using a multi-frame calculation for detecting speckle variation over time [7]. The principles of speckle-variance angiography and the preprocessing steps of fundus vessel tracing use local gradients to enhance the contrast of tubular structures and is interpreted in 2D to correlate with traditional FA imaging. However, the methods of reflectance imaging and fluorescein angiography cannot resolve both the localization of contrast agents and the depth of the vessels as tubular structures, leaving OCTA with clear advantages, in the determination of blood flow activity and vessel topography [17,18].

**Figure 1:**
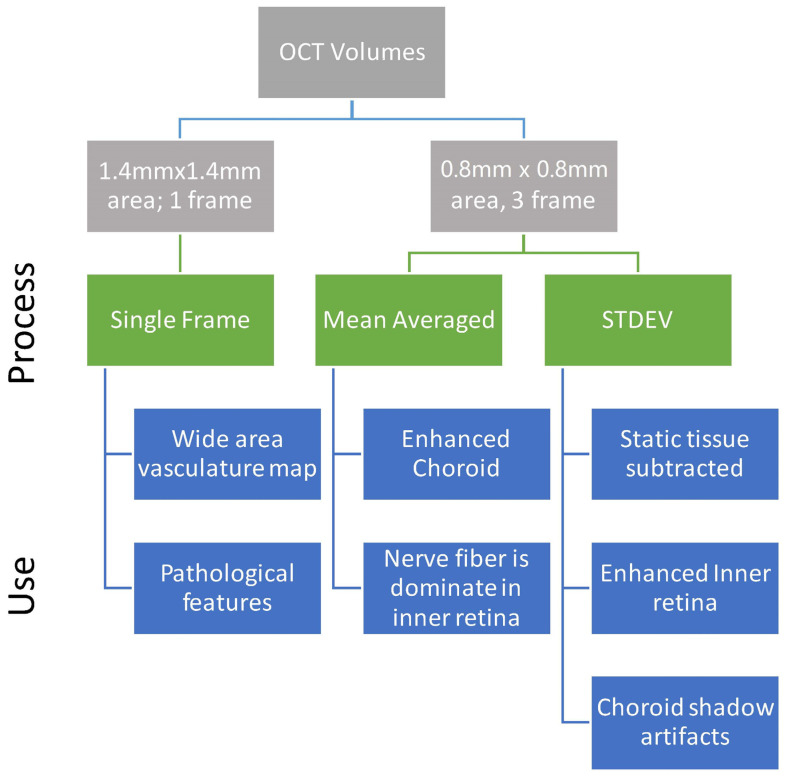
Processing scheme illustrating scan patterns and the potentially extracted information. The top levels (gray) are the OCT volumes and scan parameters used to input to the OCTA algorithm. The midlevel (green) is the processing choices, with the features highlighted listed below (blue).

Therefore, we hypothesized that by combining the theory and processing of fundus vasculature imaging in a computationally efficient 2.5D manner, the 3D vessel structure can be reliably extracted from a single en-face frame. While the temporal aspect of frame averaging is usually ignored, in this case the noise it cannot remove becomes the OCTA signal. Using the inter-scan time as a ‘slow shutter’, the flow is blurred in the volumetric scan; then, by registering for bulk motion, we use our algorithm to selectively enhance the motion blur into vascular signal. Through the implemented methodology (Figure 1), we have developed a method to detect the relationship of structural and functional changes in the microvascular network in the inner murine retina, which are important for detection and characterization of various pathologies [19,20,21] and cannot be resolved in 3D by traditional angiography methods. The novelty of this algorithm is that the spatial-temporal relationship optimizes the tradeoffs in speed and resolution, and can be optimized to use 1 frame, or averaging of as low as 3 frames, to provide the sensitivity needed to detect and characterize microvasculature. As a proof of concept, this algorithm relies only on established methodologies from fundus image vessel enhancement. The algorithm is not tied to any specific hardware, and therefore, it can operate on any OCT volumetric data with sufficient resolution, making it adaptable for many users with data acquired from different systems and organs.

## 2. Materials and Methods

To extract OCTA data from the intensity image, a near-isotropic high-density scan is required. For smooth transitions, the C-scan needs to be isotropic in the lateral plane, i.e., the same number of A-scans per both axes in the C-scan. In this experiment, the OCT was performed using an Envisu R2200 sdOCT System (Bioptigen, Durham, NC, USA) with the appropriate mouse retinal lens. With the mouse retinal lens, the native system resolution was 1.9 μm axial, and 1.4 μm lateral, 850 nm central wavelength.

**Figure 2:**
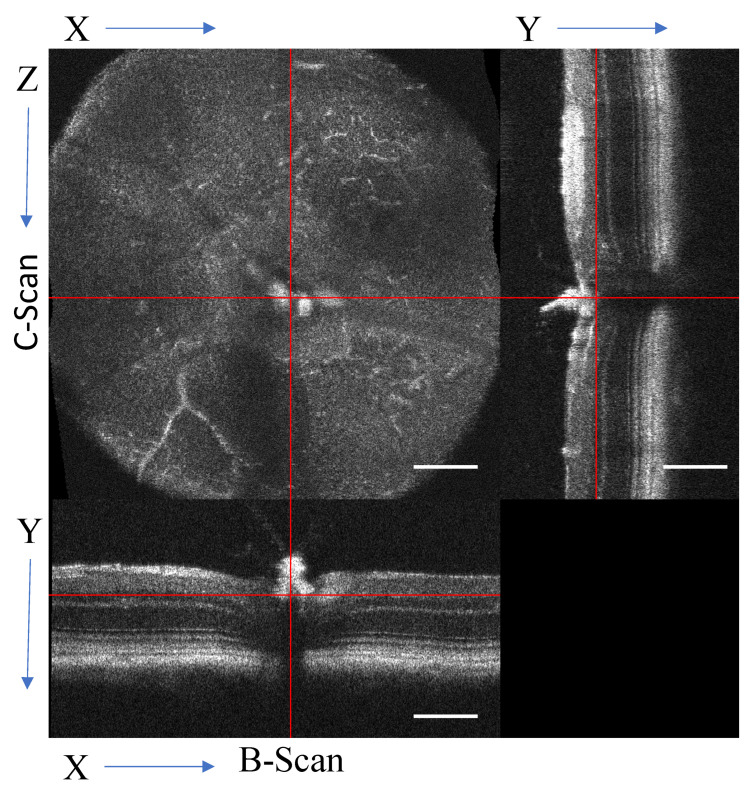
Orthographic view of the digital re-slicing of the volume for data processing. The original B-scan view [x,y,z], where the y-axis represents the A-scans that form the linear B-scan with the x-axis, and the z-axis is the sequential B-scans. The volume is transformed into the C-scan [x,z,y]. From the transformed volume, a virtual B-scan can also be generated [z,y,x]. Scale bar 200 μm.

Briefly, to prepare the data, an automated image processing routine was developed in ImageJ to handle the raw image data provided by the Bioptigen OCT system and generate the registered 3D data cube. Image registration is a critical step to minimize bulk motion and imaging artifacts [22]. The data cube was digitally resliced (Figure 2) and projected as necessary to produce the required data structure for the angiography algorithm. Optimization of the convolution kernels for processing was completed in MATLAB r2018a (MathWorks, Natick, MA, USA). For wider distribution of this prototype of algorithm, all further image processing was performed on the OCT intensity data in ImageJ v1.53e (National Institutes of Health, Bethesda, MD, USA). For open-source compliance, established algorithms and processing steps were utilized wherever possible; therefore, this algorithm is published under the Apache-2.0 license.

### 2.1. Image Acquisition Parameters and Pre-Processing

For consistency, all coordinate systems will be referenced to the raw data format with the matrix indices labeled [x,y,t,z] (Figure 2), where x is the number of A-scans per B-scan, y is the A-scan, t is the frames (same repeated B-scan), and z is the number of B-scans in the volume. Widefield OCTA was achieved by using a scan density of 1000 × 1000 A-scans, with a lateral resolution (x and z-axis) of 1.4 μm, as a single volume. Axial resolution (y-axis, depth) of the system is 1.9 μm, so the resulting data cube of the scanned volume has near isotropic resolution. Considering the exposure triangle of acquisition time, scanning area, and resolution, averaging of multiple consecutive scans (matrix index t) requires a decrease in area to a smaller region of interest; therefore, the scans were optimized for either a wide view with a single scan or a smaller view with repeated scans.

The OCT volume was optimized for a single frame and larger area 1.4 mm × 1.4 mm [x = 1000, y = 1024, t = 1, z = 1000] or for three repeated frames and 0.8 mm × 0.8 mm [x = 572, y = 1024, t = 3, z = 572]. With the Bioptigen system, the scanning rate acquired 1 million A-scans in 34 s; therefore, the imaging duration is sufficiently short for the acquisition of multiple regions or scan parameters without losing fixation or corneal clarity. On the other hand, the scanning rate is slow enough, compared to the average mouse heart rate of ~160 BPM under sedation with ketamine [23], such that temporal effects from blood flow can be exploited to yield contrast with just a single frame. With the c-scan completed in 34 s, bulk motion of breathing in the 55–65 BPM range [24] can be compensated with frame registration and bandpass filtering [22]. The acquisition speed can be improved with SS-OCT; however, the flow rate needs to travel long enough to produce speckle contrast and would be missed if scan speeds exceeded flow rate in capillaries. Adaptive optics OCTA as proposed in Salas et al. [25] can utilize the increased resolution to take advantage of both SS-OCT and our algorithm to overcome these design limitations and image microcapillaries in humans which are 5–10 μm in diameter [26,27].

With a single high-density volume, post-processing can produce multiple outputs, including high-resolution B-scans and en-face OCTA. All volumes were registered to minimize bulk motion between B-scans with ridged frame registration (StackReg plugin [28]). The repeated B-scans were frame averaged by an arithmetic mean (mean µ_t_) or standard deviation (STDEV, σ_t_) to reduce dimensionality to [x,y,z]. Once the data were in a registered 3D data cube, it was resliced (Figure 2) to the en-face view for OCTA processing following the outline in Figure 1.

### 2.2. The En-Face OCTA Algorithm

In the en-face view, the vasculature is extracted using peak-detecting strategies akin to fundus vasculature tracing. Both the structure of large vessels and the blood flow in all vessels act as a contrast agent for the vasculature, so microvasculature that has no flow will either not appear or be fragmented. This algorithm works by exploiting the scanning delay between B-scans to capture the flicker in the resliced C-scan (i.e., the spatial-temporal relationship of the speckle variance) to enhance the localized contrast in the en-face image. The resulting volume is a vessel enhanced image suitable for visualizing slice-by-slice, rendering in 3D, or projecting into optical slabs.

**Figure 3:**
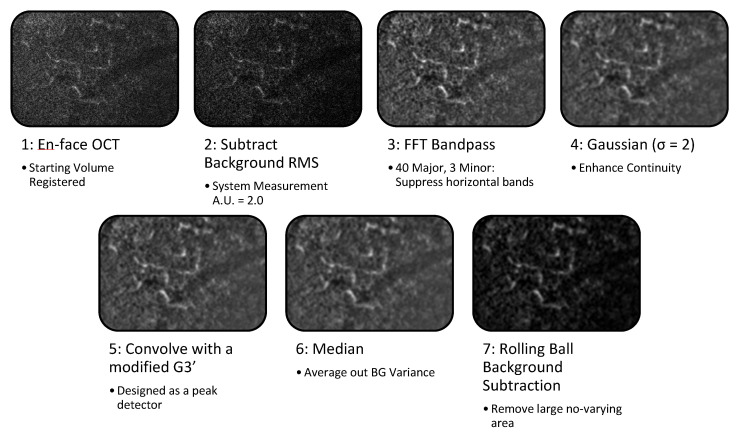
En-face ST-OCTA algorithm on ROI of a single slice. A small region was selected to show the intermediate vessel in the inner retina. Each step of the processing enhanced the localized contrast and continuity of the vessels. RMS—root mean square, FFT—Fast Fourier Transform, G3’—3rd-derivative Gaussian kernel, BG—background.

The algorithm follows the six steps outlined in Figure 3, and runs on each slice of the en-face volume. The data processing utilizes the 2.5D approach [29] by applying 2D image processing for peak and edge detection to each slice of a 3D volume. The 2.5D approach takes the volume [x,z] plane to enhance the vasculature, and then enhances continuity between the slices [y] axis. The following parameters were optimized for robust vessel enhancement at from 5 µm to 40 µm without needing multiple scale operations. First, from the en-face OCT, the background that was measured as the root mean squared (RMS) was subtracted as a constant offset; in our system, the RMS grayscale value of non-tissue was 20 a.u. in the 8-bit image. For use with other systems, this is the only parameter that will require alteration for detecting vessels with a diameter of 2–20 voxels. Secondly, the high- and low-frequency banding caused by bulk motion, such as the mouse’s breathing rate (~1–4 Hz), the heart rate (~5–13 Hz), and registration errors was compensated for with a bandpass filter with horizontal stripe suppression [29,30]. With the bulk motion, the periodicity of the breathing rate was major and minor scales were 40 and 3; frame averaging of 3 did not alter the scale nor did the changes in heart rate and breathing under anesthesia, so when combined with frame registration, this parameter should be robust for various imaging conditions. Through the 2.5D processing and registration, the bulk motion resulted in a contiguous frame registration error while blood flow results in the localized “flicker”. The third step was to improve the continuity of the image gradients using a Gaussian with a σ = 2 scaling, σ is discrete Nyquist criteria for capillary size of 5 μm. This provides the capability to robustly detect vessels with a diameter of 4 voxels in all 9 discrete orientations. The fourth step is ridge enhancement, with a modified 2D peak detector based on a third-derivative Gaussian kernel (G3’) scaled for detecting the microvasculature. The Gaussian kernel is truncated with a zero-padding and a notch for rotational variance. In the design of the 2D filter, it was specifically crafted to use the 3rd derivative Gaussian to enhance vesselness, furthermore the std projection is another way to enhance variance over time and space. The fifth stage is a median filter to suppress errors in the ridge detection caused by background variance. The final stage is to use a rolling ball background subtraction algorithm as an adaptive localized method to remove large areas of non-varying background intensity of the static tissue.

When all stages are complete, the tubular structures of the en-face view are enhanced in contrast from the static tissue, and more continuous than the raw data. The 2.5D process outputs a vessel-enhanced volume with the coordinates system co-registered with the original C-scan. The alternative processing and visualization outputs may be adapted to suit the varying imaging conditions.

### 2.3. Visualizing the Angiography: 3D and Projection Selection

**Figure 4:**
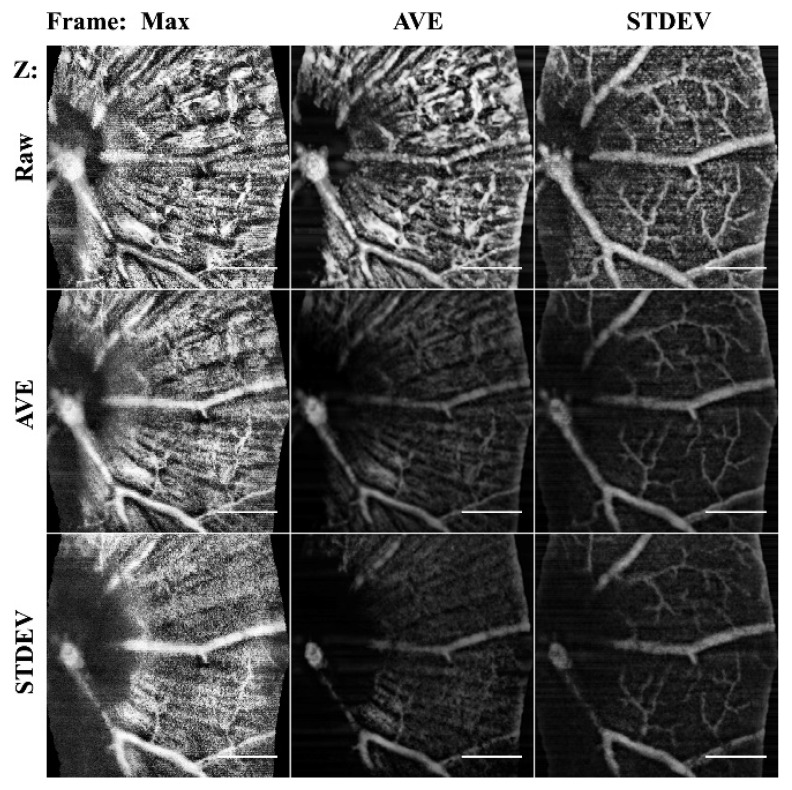
Comparison of frame averaging methods. Columns are the frame averaging method used to project the 60 μm stack, and the rows are the input b-scan averaging method used. By altering the combinations, the specificity to detect different the morphology is selectable. STDEV frame averaging improved vessels, where mean AVE enhanced NFL. MAX intensity projections only captured the ILM. Scale bar 200 μm.

The core algorithm was used to enhance the vascular structure. The initial data processing was chosen to optimize the physiology of interest. Projection methods alter the data to reduce dimensionality, therefore an understanding of the changes of the structures enhanced or eliminated is needed. While a maximum (max) or average (ave) projection methods are most commonly used to reduce variance and noise in the image, in angiography data they remove signal, therefore, standard deviation (STDEV) are proposed to increase visibility of vessels as their speckle noise increases variance. Through projections, the data were reduced from 4D (x,y,t,z) into 2D color mapped images (x,y,color). In Figure 4, the STDEV frame averaging enhanced vasculature in all cases. B-scan averaging improved the specificity of the NFL or vasculature.

**Figure 5:**
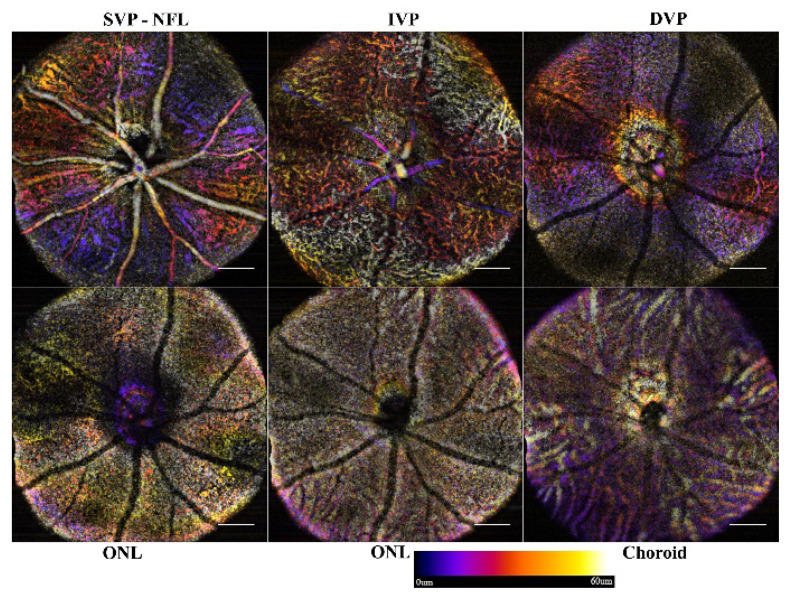
Sample data of single frame ST-OCTA depth projections. Each 60 μm slab was projected using a color-coded depth map. Starting from the inner limiting membrane, each consecutive slab (6 in total for normal pathology) was generated until the choroid was reached. Scale bar 200 μm.

To visualize the angiogram, the data can be reviewed slice-by-slice through the volume, rendered as a 3D object, or projected with color-coded depths. Currently, the OCT slab requires manual selection to define the starting limit for projections. Orthographic projections, as used as in Figure 2, are used to select slabs for analysis. For consistency in data analysis, a routine was written to start from the selected slice, and project consecutive 60 μm slabs through the end of the stack, Figure 5. The color-coded projection provides depth information of the vascular beds with blue being superficial, reds ~30 μm deep, and yellow-white being 60 μm.

Visualization as a 3D object of the initial data or vessel enhanced images was performed with the ImageJ 3D viewer plugin. Image stacks were first binarized with an adaptive thresholding algorithm based on local first-order statistics. In the rendering options, the “surface plot” transform was chosen conversion method to export from the 3D Viewer plugin as a STL file format. Final rendering and merging of the resulting 3D volumes were performed in ImageJ.

### 2.4. Methods for Animal Care

All animal procedures were approved and performed per the UTMB IACUC regulations, NIH/NEI, and the IOVS guidelines for use of animals in ophthalmic research. All procedures were performed under injectable anesthesia (ketamine/dexmedetomidine). Eyes were dilated using a serial application of proparacaine, phenylephrine, and tropicamide (Alcon). Corneal hydration was maintained with lubricating drops (Alcon). In the studies, 11 exemplar mice, without any discernable pathologies, were used for performance analysis.

## 3. Results and Discussion

Through the algorithm presented, tubular objects were reliably detected. With the sampling resolution, the vessels in the inner retina were detected in the separate microvascular beds. The enhanced views allowed choroidal vascular imaging.

### 3.1. Single Frame Widefield: Assessment of the Algorithm

The 1.4 mm field of view provides the ability to scan a sufficient area to cover the optic nerve head (ONH) and the vasculature surrounding it. With a single frame, the full scan was acquired in 34 s and, with stack registration before reslicing, the motion artifacts were minimized. The remaining bulk motion (i.e., motion due to respiration and pulse) was compensated for with the bandpass filter, allowing clear resolution of the nerve fiber layer (NFL), intermediate vascular plexus (IVP), and deep vascular plexus (DVP). With the single frame view, a wide area can be imaged rapidly and the microvasculature of the IVP and DVP visualized. The microvasculature of the superficial vascular plexus (SVP), however, cannot be distinguished from the NFL. An example of a naive or defect-free retina of an adult mouse is shown in Figure 5. Through the layers, the vasculature can be well established. The ONL-containing slabs show no vasculature or intrusions; however, the shadowing artifacts of the superficial vasculature are clearly visible.

### 3.2. Frame Averaging by Mean Approach

**Figure 6:**
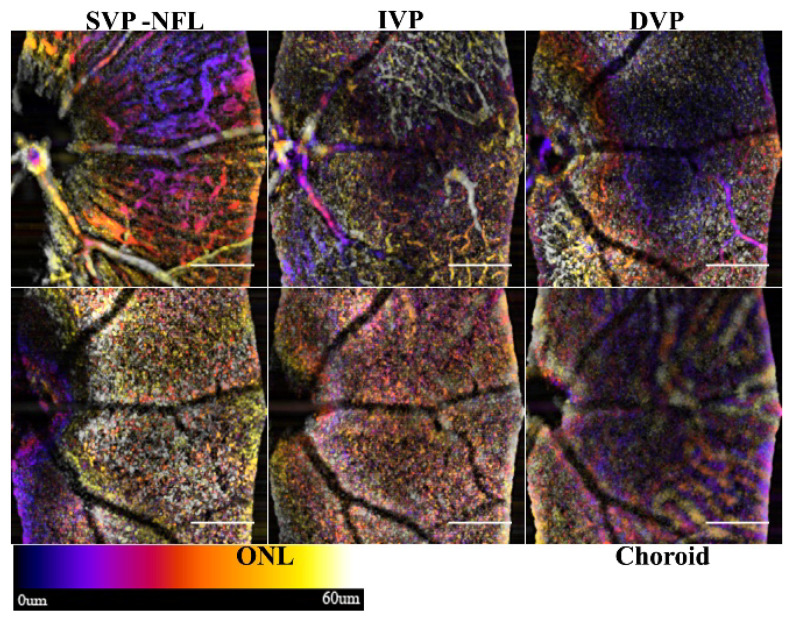
Sample retina showing NFL and choroid enhancement by frame averaging. The three frames were averaged (µt) and processed with the angiography algorithm. The nerve fiber bundles and choroid are enhanced in the SVP and NFL. Scale bar 200 μm.

Averaging the intensity values of three or more consecutive frames at the same position is the traditional way to limit speckle variation and improve the estimated signal to noise ratio (SNR) or Contrast to Noise Ratio (CNR). This variation of the processing is typically employed to improve the SNR in the B-scans, but, for OCTA, the sensitivity for detecting nerve fibers and choroid vessels is enhanced (Figure 6). The SVP and NFL in Figure 6 are enhanced with more pronounced edges when compared to the single frame view in Figure 5. 

Both the NFL and choroid contain large tubular structures, whose static component of the signal can be detected as a ridge. The utility of enhancing the NFL is that neurodegeneration and the contour of the inner retinal layers can be assessed. The NFL slab of the angiography shows the nerve bundles that correlates to traditional red-free fundus imaging. The microvasculature in the SVP, however, is obscured by the nerve bundles.

Frame averaging can improve image quality; however, the longer dwell time and smaller area assessment make it prone to motion artifacts. A second registration step may be required if motion drift is not fully compensated for in the initial registration.

### 3.3. Frame Averaging by Standard Deviation

**Figure 7:**
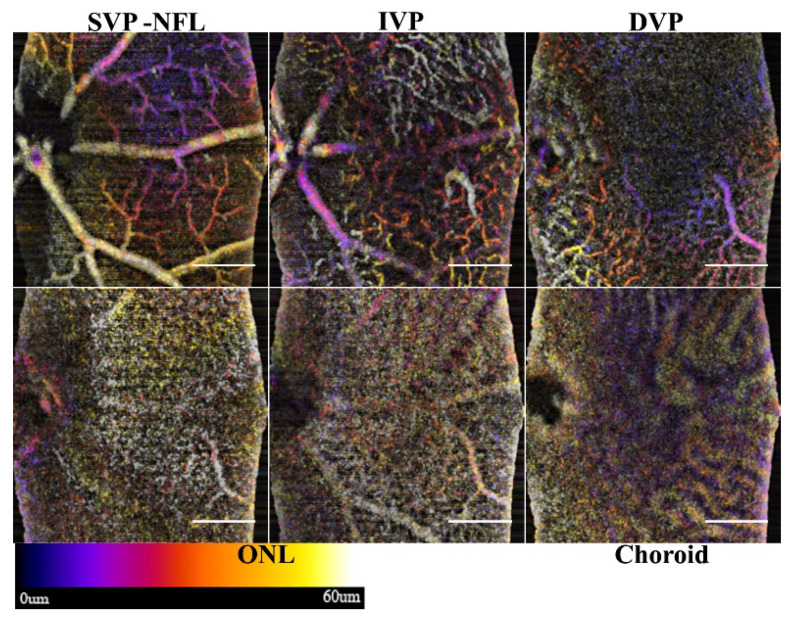
Enhanced microvasculature through STDEV frame averaging. The alternative processing of the same volume used to generate Figure 6 shows the increased specificity of the microvasculature. The SVP, IVP, and DVP are enhanced, showing all of the microvasculature. The ONL and Choroidal slabs show artifacts of the superficial vasculature. Scale bar 200 μm.

The standard deviation of the frames is, by transitive properties, the complex signal consisting of phase variance and speckle variation. Instead of decorrelation, however, the STDEV is computed during frame averaging, enhancing the specificity of the algorithm to see small flowing vessels in the inner retina. However, static tissue features, such as nerve fiber bundles, have lower variance compared to the turbidity of flowing fluids. Unlike the mean averaging method, the proposed algorithm rejects the static component of the NFL and choroid. Through this flow enhanced discrimination, the inner plexiform layer improves the intermediate and micro-vasculature that can be resolved although the flow in the choroid the signal is decreased. Figure 7 shows the same retina as in Figure 6; however, the visualization of the vasculature of the inner retina is enhanced.

The frame averaging increases the specificity of the OCTA algorithm, and explicitly differentiates SVP microvasculature over the nerve fiber bundles in the NFL. The shadow artifacts in the ONL are inverted and could lead to false positives.

### 3.4. Comparisons of Averaging Methods

While Figure 5 and Figure 6 offer a qualitative comparison of the B-scan averaging methods, the improvements to the specificity can be measured more directly as the contrast-to-noise ratio (Equation (2)). Line profiles and analysis of the measured CNR were used to validate the improvement in specificity in the three vascular plexuses (superficial, intermediate, and deep).

**Figure 8:**
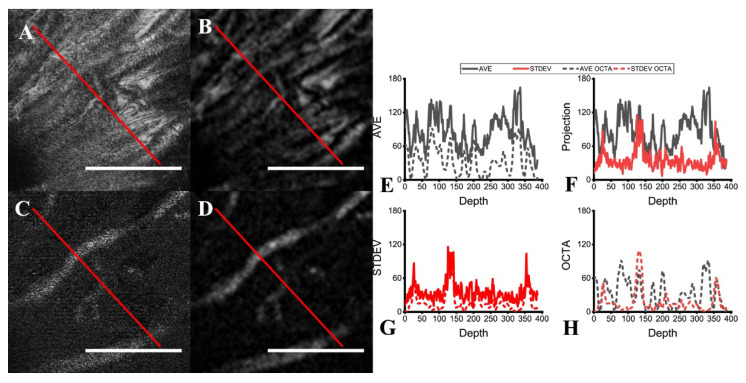
Comparison of mean and STDEV frame averaging. Lines were drawn over the projections of the same retinal slice with (**A**) mean and (**C**) STDEV frame averaging, as well as the corresponding OCTA slice (**B**,**D**), for line intensity profile measurement (red line). The line profiles for the projections and OCTA for (**E**) mean averaging and for (**G**) STDEV averaging are shown. Comparisons between the two averaging methods’ line profiles are also shown for both (**F**) the projections and (**H**) the OCTA slice. Scale bar 200 µm.

Figure 8 compares the two frame averaging methods with line profiles across the same retinal slice as either a projection or an OCTA image. The improved specificity of the STDEV frame averaging towards flowing features (i.e., vessels) is clearly visualized (Figure 8C,D), while stationary features (e.g., nerves, fiber layers) are detected as well by mean frame averaging (Figure 8A,B). In the line profiles, the vessels manifest as distinct peaks in STDEV frame averaging (Figure 8G), while the fibers present in the same layer appear as a pattern of ridges (Figure 8E). Through the line profiles in Figure 8H, the specificity of the STDEV averaging method is evident, as the nerve fiber bundles are rejected. As each post-processing routine is run on the same scan, this provides fixability in the contrast enhancement for data analysis depending on the structures to be isolated.

### 3.5. Performance Comparison

We used the same mouse retinal image stack (571 × 571 × 1024) captured by our imaging system to compare the performance of different algorithms. Preprocessing, namely image registration to correct for bulk motion, was done to prepare the dataset prior to application of each algorithm. The algorithms evaluated were speckle variance [31], phase variance [32], and complex differential variance [33], in comparison to our single frame ST-OCTA algorithm and multi-frame standard deviation-based ST-OCTA algorithm. All algorithms were tested on a laptop PC with a quad-core Intel i7 (6th gen) 6700HQ with 32 GB of 2133 MHz DDR4 RAM.

We recognize that it is difficult to compare OCTA algorithm performance, due to lack of a ground-truth vasculature map for in vivo studies. To compensate, we followed the procedure described by Zhang [34], and averaged the image stacks generated by all of the algorithms tested with equal weight given to each [19]. The averaged image stack was used to create a vascular mask that was used as ground truth, using a modification of the method described by Reif [35], in which a low-pass filter was applied to remove small elements, followed by a global threshold to set all pixels below it to zero, and finally a local adaptive threshold to binarize the image [35]. Instead of skeletonizing the binarized image as they did, we used the binarized image directly as a vessel mask, as our vasculature had a significantly larger pixel width [35]. The estimate of the signal-to-noise ratio (SNR) was then calculated using the vasculature mask to determine the signal and the background intensities, using the equation:

(1) $$\mathrm{SNR}=\frac{\mathrm{Mean}\left(\mathrm{signal}\right)}{\mathrm{Std}\left(\mathrm{background}\right)},$$

where Mean(signal) indicates the mean value of intensities on the vessel mask and Std(background) indicates the standard deviation of intensities on the inverse of the vessel mask [34].

In Table 1, the SNR achieved by our algorithms is shown to be greater than those used for our comparison analysis, with shorter computation times, as well (12.39 s for single-frame and 14.49 s for multi-frame), except against the original speckle variance algorithm (6.05 s). Phase variance was the slowest algorithm due to the calculations of phase angle, and had the worst SNR. On closer examination of the resultant image stack, every three to four images consistently had drastically reduced signal, likely due to bulk motion that was unable to be compensated for by our image registration step prior to phase variance, demonstrating a weakness of that algorithm.

**Figure 9:**
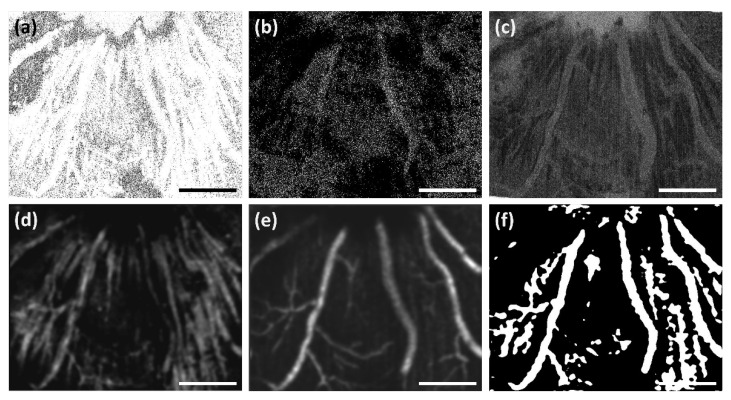
Performance comparison of OCTA algorithms. Retinal blood vessels visualized by (**a**) speckle variance, (**b**) phase variance, (**c**) complex differential variance, (**d**) single-frame ST-OCTA, (**e**) multi-frame ST-OCTA (STDEV), and (**f**) the estimated ground truth. Scalebar 200 µm.

It should be noted that due to the tendency of some algorithms to pick up non-vascular tissue features, particularly at the NFL (Figure 9), the estimated ground truth vasculature map is likely inaccurate at that depth, affecting SNR reliability. For example, the speckle variance and our single frame algorithms pick up a significant amount of connective tissue, visible in Figure 9, compared to the multi-frame standard deviation-based variant. Furthermore, some algorithms generate more shadow artifacts (e.g., speckle variance, complex differential variance), potentially causing them to be misidentified as part of the ground truth vasculature map in deeper layers.

### 3.6. CNR Performance Metrics

**Figure 10:**
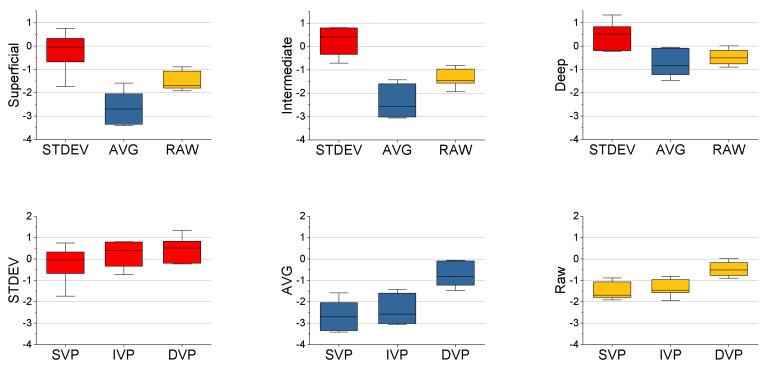
Comparison of CNR by vascular plexus and frame averaging method (n = 7). Boxplots of the CNR of the vascular plexuses separated by frame averaging methods (top), showing the highest CNR with the STDEV averaging, followed by the raw images, and finally the mean averaging. Boxplots for the CNR of the frame averaging methods were plotted as well (bottom), displaying the highest CNR in the deep vascular plexus, followed by the intermediate and superficial vascular plexuses. For all conditions, both paired t-test and ANOVA showed significant differences (*p* < 0.05).

Because there are not currently any standardized benchmark performance metrics for comparing between systems and methodologies, we are providing a template for CNR calculations at different physiological layers. As the SNR in tissue is estimated, the CNR is a more robust way to validate the measurements. The CNR was calculated (n = 7) at each vascular plexus (i.e., superficial, intermediate, deep), and for each frame averaging method (i.e., STDEV, mean, raw). The CNR (Equation (2)) [36] was calculated with the following equation:

(2) $$\mathrm{CNR}=\frac{\mu_{s}-\mu_{n}}{\sqrt{0.5(\sigma_{s}^{2}+\sigma_{n}^{2})}},$$

with µ_s_ indicating the mean vessel signal, µ_n_ indicating the mean background tissue noise, σ_s_ indicating the standard deviation of the vessel signal, and σ_n_ indicating the standard deviation of the background tissue noise. STDEV frame averaging had the highest CNR across the vascular plexuses, demonstrating its higher vessel specificity, while mean frame averaging had the CNR lower than even the raw images, showing its higher specificity towards non-vessel structures (e.g., nerve fibers) (Figure 10). Overall, the superficial vascular plexus had the lowest CNR, likely due to the presence of the NFL causing a higher background and increased noise, while the deep vascular plexus had the highest CNR, presumably due to the reduced influence of the NFL on the background.

**Figure 11:**
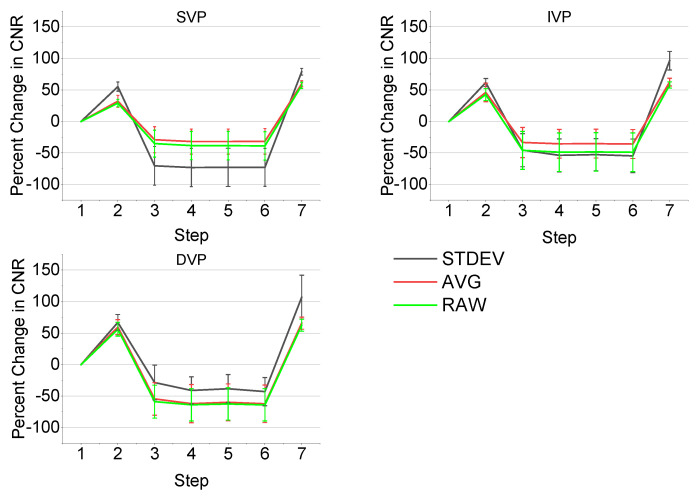
Change in CNR with each processing step by vascular plexus and frame averaging method (n = 11). The step numbers shown on the x-axis correspond to the seven steps of the algorithm shown in Figure 3.

To demonstrate the improvements provided by the algorithm in a stepwise manner, the CNR was calculated (n = 11) at each step of the en-face OCTA algorithm, as described in Figure 3 (e.g., step 1 is the starting volume after application of an averaging method, step 2 is the subtraction of background RMS, and so on). The percent change in CNR was plotted for each step and for each vascular plexus and the results are shown in Figure 11. The CNR increases at first after subtracting the background RMS, but decreases following a FFT bandpass filter. The following three steps offer no significant changes in CNR, as the goal of these steps is primarily to enhance general features and improve continuity. However, the final step, a rolling ball background subtraction, causes a dramatic increase in CNR as it is designed to correct and smooth out uneven signals across the image. Overall, the STDEV frame averaging shows the greatest CNR improvements by the end of the algorithm, which was expected due to its greater vessel sensitivity.

### 3.7. Volumetric Analysis and High-Resolution B-Scans

**Figure 12:**
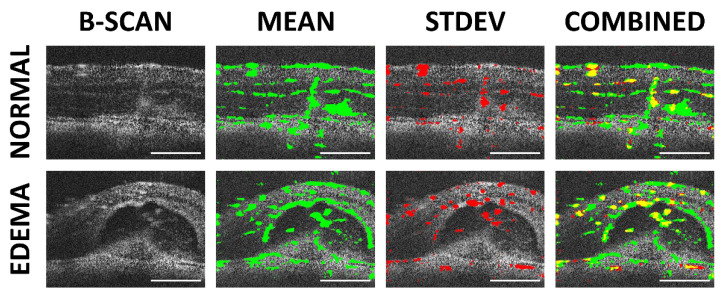
High-resolution analysis of B-scans from a normal retinal region and CNV lesion with edema. The high-resolution B-scans (far right) were overlaid with the results from mean (green) and STDEV frame averaging (red). In the combined image, the overlap between the two averaging methods is shown in yellow. Scale bar 200 μm.

After optimizing the scan parameters for OCTA, the traditional B-scans can be assessed for high-resolution analysis to correlate OCTA features to morphology, as shown in Figure 12. The two methods for frame averaging result in the emphasis of different structures: mean averaging highlights the stationary vessel-like structures (e.g., nerve fibers and choroid vessels), while STDEV averaging highlights the flowing features (i.e., blood flow). Where the two methods overlap tends to be in the region where larger blood vessels are present, as the thicker vessel walls are picked up by the mean frame averaging and the blood flow by the STDEV frame averaging. With the algorithm providing the capability to distinguish between stationary and flowing features, it may be possible, in the future, to perform vessel perfusion analysis to evaluate disease progression, such as glaucoma [19]. However, the primary limitation of this technique is that in the SVP region, completely occluded vessels may not be differentiated by OCTA.

**Figure 13:**
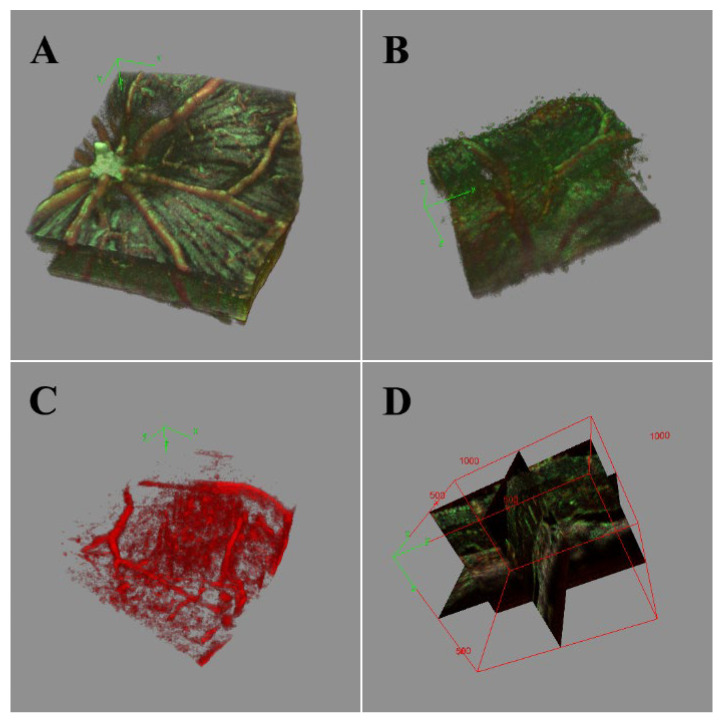
3D-rendered volume of normal retinal region (**A**) and CNV lesion with edema (**B**–**D**). The red portion of the volume shows the result of STDEV frame averaging with increased microvasculature specificity, while the green portion of the volume shows the result of mean frame averaging. (**A**) Organized NFL tracks and vascular branching. (**B**) The bulge of CNV disrupting the NFL and vasculature. The edemas are apparent in the 3D render of the STDEV vasculature (**C**) and visualized in 3D orthographic views (**D**).

From the B-scans, image stacking can be conducted to allow volumetric analysis (Figure 13). Important, layer-specific morphological characteristics, such as vessel density and tortuosity, can be examined qualitatively in the resulting image volume. Ultimately, with the increased vessel discrimination sensitivity and improved overall contrast provided by the combination of mean and STDEV frame averaging, enhanced automated vessel and lesion segmentation can be performed with existing algorithms (e.g., Frangi vesselness, multiscale Hessian analysis). This would allow for more quantitative and clinically relevant measurements of lesions, edemas, and vessel tortuosity than currently available, improving our ability to visualize and understand disease states.

### 3.8. Generalizability and Alternate Methods

While initially designed for the mouse retina, the algorithm was written to be generalizable for OCT volumes. Accordingly, we explored other processing steps (discussed as follows); our order of operations and scales were optimized to enhance edge gradients and detect vessels 2–40 voxels in diameter. Without any major alterations, we tested the algorithm’s feasibility on cornea neovascularization data, and from data acquired from other systems. We also explored how various 3D filters affected the performance of the algorithm. For example, with the corneal images, the averaged projections served as an index for measurements of the severity and area of corneal opacity, while the standard deviation isolated the neovascularization from the ghost blood vessels [37].

**Figure 14:**
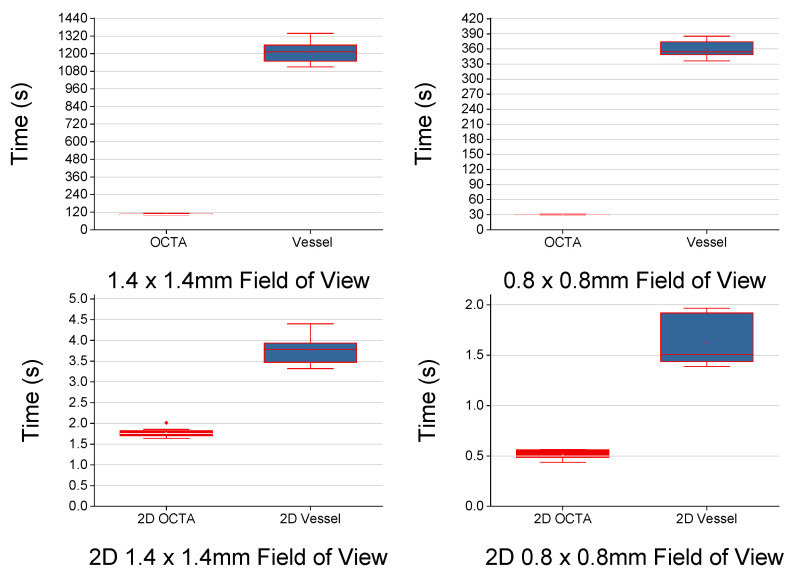
2D and 3D volume processing time. The red plots are the OCTA algorithm, and the blue plots are the vesselness step. The 3D Frangi (σ = 3) is significantly slower (10×) than the proposed algorithm, in 2D, the difference is closer to 3×. All time differences are significant.

An advantage of the 2.5D approach is that the optimized 2D filters outperform 3D filters on the en-face view. While 3D Gaussian or Median filters can replace their 2D counterparts in our algorithm, the most comparable filter to replace the notched convolution kernel is a “vesselness” filter. A majority of the existing algorithms use the Hessian or Frangi vesselness filters [38,39,40,41,42]. In our methodology, the stepwise processing reproduces many of the effects of the Hessian gradient and peak enhancing filter, though discrete local filters. In Figure 14, we show that there is a significant improvement to filtering with our proposed algorithm when compared to the Frangi filter. As noted by Rocholz et. al. [39], the Frangi implementation can introduce artifacts due to the chosen sigma scale, and additionally mis-identify shadow artifacts as vasculature. While working with 2D projection images can improve the speed, the 2D projection alters the morphology and can mask changes, which can be disadvantageous. As noted by Ploner et al., the 12 μm resolution and scan speed of the ssOCT system could not image the choriocapillaris or smallest vasculature, even with vesselness enhancement [43]. With the commercial systems used, the 3 × 3 mm FOV were indicated for imaging smaller vessels; however, even with a slower multiscale vesselness and adaptive thresholding, small vessels were cleaned (i.e., removed by thresholding) to improve the aesthetics of the angiography [44,45]. With the considerations of sensitivity/specificity and overall angiographic aesthetic, we followed Heidelberg’s example and erred on the side of presenting vessel enhanced maps with the 5–10 μm capillaries resolved, instead of thresholding them [39].

**Figure 15:**
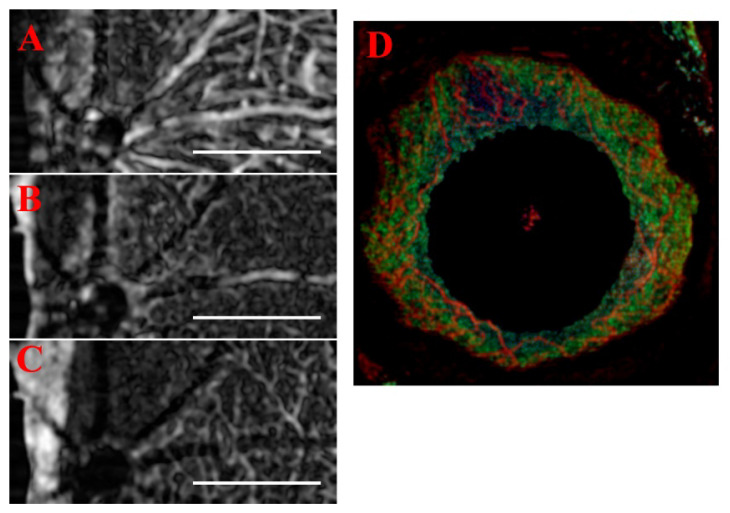
OcuScience imaging system retina and Bioptigen retina. (**A**) Superficial vascular plexus, (**B**) intermediate vascular plexus, and (**C**) deep vascular plexus imaged with OcuScience system and processed with the single frame algorithm. (**D**) Bioptigen 18 mm lens to image anterior segment, without pathology, the cornea shows no vasculature, and the iris vasculature is resolved. Scale bar 200 μm.

As the prototype ST-OCTA algorithm was developed for the retina, the generalization was tested using data from another system and for anterior segment OCT. The single frame algorithm results were replicated using data from the OcuScience iVivo^®^ LAB OCT system at 3 µm resolution and the three vascular plexus layers were resolved (Figure 15A–C). The only required modification was using the 3D median [5 × 5 × 3] in place of the 2D Gaussian. While the 3D median is slower than the 2D Gaussian, a slight cost of the overall impact on processing time is an acceptable tradeoff for robustness and generalizability to use data from other OCT systems. Furthermore, the Bioptigen system’s anterior chamber 18 mm lens was used to image the iris vasculature with a 5.2 µm resolution, using the 3-frame method and compositing the methods (Figure 15D). The standard deviation projection method provided the clearest visualizations of both the iris vasculature and the mean projection of the opacity scoring system. When applied in a toxicology model, both the iris vasculature and corneal neovascularization was analyzed using the OCTA algorithm without modification, and the 3D data was projected in 300 µm slabs to compare vascular distributions against FA imaging [37,46]. Similar to the data illustrated in Figure 15, application of this approach to assess the response of mouse cornea to topical administration of an ocular toxicant allowed for quantifiable characterization of neovascularization and corneal opacity as a function of time after toxin exposure.

Currently, the prototype ImageJ algorithm is available on Github at https://github.com/UTMB-Luisi/Spatial-Temporal-Speckle-Variance-OCTA (21 February 2022). In the future, we plan to develop the algorithm towards easing the difficulty of automated 3D segmentation, by reducing the processing complexity through the 2.5D methodology. The spatial-temporal relationship and 2.5D processing optimize the efficiency by allowing the application of established vessel segmentation algorithms to the OCT image volumes, as well as optimization of the overall algorithm for improved computation speed through slice-by-slice parallelization on a GPU.

## 4. Conclusions

As the adoption of OCTA in the clinic continues, there is a growing need for robust and efficient algorithms to increase the prevalence of this technique in both pre-clinical and clinical studies. OCTA, as a label-free angiography modality, can identify the layer-specific changes in the microvasculature of ocular tissue. In this paper, our algorithm exploits the fact that spatial and temporal aspects of the scanning process are indivisibly interlaced. While the convolution kernel can enhance motion blur into vesselness from a single frame, the 3-frame projection methods further increase our specificity and provided the greatest CNR. The main advantages of our proposed algorithm are its generalizability to different systems, its relative speed, its simplicity in its use of common image processing methods, its low computational requirements, and that it is publicly available/open source. The ST-OCTA algorithm may not be as effective as some proprietary algorithms that are system-specific, and its use would have the most significant benefit with SD-OCT systems or systems that have a relatively slow scanning speed.

The novel algorithm presented here improves upon traditional OCTA by removing the need for computation-heavy decorrelation algorithms to detect vasculature. Furthermore, the algorithm can be adapted for any en-face OCT data where the sampling density approaches the Nyquist criterion for visualization of the microvasculature. The algorithm provides unique flexibility, including the capability of using a single frame or multiple frames, depending on the desired target. Specifically, our approach using a single frame is adequate for the visualization of all layers of the retina except for the superficial layer, where frame averaging is required to reduce the artifacts introduced by the nerve fiber layer. With the contrasting specificity for flowing and stationary features offered by the two complementary frame-averaging methods, the algorithm presented here offers great potential for improving our understanding of retinal diseases and their progression.

## Figures and Tables

**Table 1:** Comparison of OCTA algorithms. Note: SV, speckle variance; PV, phase variance, CDV, complex differential variance; SF ST-OCTA, single-frame ST-OCTA; MF ST-OCTA, multi-frame ST-OCTA.

Parameter	SV	PV	CDV	SF ST-OCTA	MF ST-OCTA
Signal-to-Noise Ratio	0.235	0.0817	0.168	0.304	0.381
Computation Time (s)	6.05	170.37	23.17	12.39	14.49

## Data Availability

The authors confirm that the data supporting the findings of this study are available within the article. The ST-OCTA algorithm files are available on Github at https://github.com/UTMB-Luisi/Spatial-Temporal-Speckle-Variance-OCTA (21 February 2022).

## References

[1] Huang D., Swanson E.A., Lin C.P., Schuman J.S., Stinson W.G., Chang W., Hee M.R., Flotte T., Gregory K., Puliafito C.A. (1991). Optical Coherence Tomography. Science.

[2] Alonso-Caneiro D., Read S.A., Collins M.J. (2011). Speckle reduction in optical coherence tomography imaging by affine-motion image registration. J. Biomed. Opt..

[3] Mayer M.A., Borsdorf A., Wagner M., Hornegger J., Mardin C.Y., Tornow R.P. (2012). Wavelet denoising of multiframe optical coherence tomography data. Biomed. Opt. Express.

[4] Jia Y., Bailey S.T., Wilson D.J., Tan O., Klein M.L., Flaxel C.J., Potsaid B., Liu J.J., Lu C.D., Kraus M.F. (2014). Quantitative Optical Coherence Tomography Angiography of Choroidal Neovascularization in Age-Related Macular Degeneration. Ophthalmology.

[5] Ang M., Cai Y., Shahipasand S., Sim D.A., Keane P.A., Sng C.C.A., Egan C.A., Tufail A., Wilkins M.R. (2015). En face optical coherence tomography angiography for corneal neovascularisation. Br. J. Ophthalmol..

[6] Jia Y., Tan O., Tokayer J., Potsaid B.M., Wang Y., Liu J.J., Kraus M.F.G., Subhash H., Fujimoto J.G., Hornegger J. (2012). Split-spectrum amplitude-decorrelation angiography with optical coherence tomography. Opt. Express.

[7] Choi B., Kang N.M., Nelson J. (2004). Laser speckle imaging for monitoring blood flow dynamics in the in vivo rodent dorsal skin fold model. Microvasc. Res..

[8] Liu Q., Chen S., Soetikno B., Liu W., Tong S., Zhang H.F. (2017). Monitoring Acute Stroke in Mouse Model Using Laser Speckle Imaging-Guided Visible-Light Optical Coherence Tomography. IEEE Trans. Biomed. Eng..

[9] Mahmud M.S. (2013). Speckle Variance Optical Coherence Tomography (svOCT).

[10] Shi W., Gao W., Chen C., Yang V.X.D. (2017). Differential standard deviation of log-scale intensity based optical coherence tomography angiography. J. Biophotonic.

[11] Wang J., Zhang M., Hwang T.S., Bailey S.T., Huang D., Wilson D.J., Jia Y. (2017). Reflectance-based projection-resolved optical coherence tomography angiography. Biomed. Opt. Express.

[12] Lee P.-H., Chan C.-C., Huang S.-L., Chen A., Chen H.H. (2018). Extracting Blood Vessels From Full-Field OCT Data of Human Skin by Short-Time RPCA. IEEE Trans. Med. Imaging.

[13] Le N., Song S., Zhang Q., Wang R.K. (2017). Robust principal component analysis in optical micro-angiography. Quant. Imaging Med. Surg..

[14] Spaide R.F., Klancnik J.M., Cooney M.J. (2015). Retinal Vascular Layers Imaged by Fluorescein Angiography and Optical Coherence Tomography Angiography. JAMA Ophthalmol..

[15] Hormel T.T., Wang J., Bailey S.T., Hwang T.S., Huang D., Jia Y. (2018). Maximum value projection produces better en face OCT angiograms than mean value projection. Biomed. Opt. Express.

[16] Liu W., Luisi J., Liu H., Motamedi M., Zhang W. (2017). OCT-Angiography for Non-Invasive Monitoring of Neuronal and Vascular Structure in Mouse Retina: Implication for Characterization of Retinal Neurovascular Coupling. EC Ophthalmol..

[17] De Carlo T.E., Romano A., Waheed N.K., Duker J.S. (2015). A review of optical coherence tomography angiography (OCTA). Int. J. Retin. Vitr..

[18] Chen C.-L., Wang R. (2017). Optical coherence tomography based angiography [Invited]. Biomed. Opt. Express.

[19] Yang Y., Wang J., Jiang H., Yang X., Feng L., Hu L., Wang L., Lu F., Shen M. (2016). Retinal microvasculature alteration in high myopia. Invest. Ophthalmol. Vis. Sci..

[20] Bhanushali D., Anegondi N., Gadde S.G.K., Srinivasan P., Chidambara L., Yadav N.K., Roy A.S. (2016). Linking retinal microvasculature features with severity of diabetic retinopathy using optical coherence tomography angiography. Investig. Ophthalmol. Vis. Sci..

[21] Wakabayashi T., Sato T., Hara-Ueno C., Fukushima Y., Sayanagi K., Shiraki N., Sawa M., Ikuno Y., Sakaguchi H., Nishida K. (2017). Retinal microvasculature and visual acuity in eyes with branch retinal vein occlusion: Imaging analysis by optical coherence tomography angiography. Investig. Ophthalmol. Vis. Sci..

[22] Xiao M., Zou C., Sheppard K., Krebs M. OCT Image Stack Alignment: One more important preprocessing step. Proceedings of the BioImage Informatics Conference 2015.

[23] Ewald A.J., Werb Z., Egeblad M. (2011). Monitoring of Vital Signs for Long-Term Survival of Mice under Anesthesia: FIGURE 1. Cold Spring Harb. Protoc..

[24] Ho D., Zhao X., Gao S., Hong C., Vatner D.E., Vatner S.F. (2011). Heart Rate and Electrocardiography Monitoring in Mice. Curr. Protoc. Mouse Biol..

[25] Salas M., Augustin M., Ginner L., Kumar A., Baumann B., Leitgeb R., Drexler W., Prager S., Hafner J., Schmidt-Erfurth U. (2016). Visualization of micro-capillaries using optical coherence tomography angiography with and without adaptive optics. Biomed. Opt. Express.

[26] Rha J., Jonnal R.S., Thorn K.E., Qu J., Zhang Y., Miller D.T. (2006). Adaptive optics flood-illumination camera for high speed retinal imaging. Opt. Express.

[27] Weinhaus R.S., Burke J.M., Delori F.C., Snodderly D.M. (1995). Comparison of fluorescein angiography with microvascular anatomy of macaque retinas. Exp. Eye Res..

[28] Thevenaz P., Ruttimann U., Unser M. (1998). A pyramid approach to subpixel registration based on intensity. IEEE Trans. Image Process..

[29] Sherfuddin M., Vijay K., Prashanthi H.M.G., Sathya G. Skeletonization of 3D Images using 2.5D and 3D Algorithms. Proceedings of the 2015 1st International Conference on Next Generation Computing Technologies (NGCT).

[30] Jia Y., Wei E., Wang X., Zhang X., Morrison J.C., Parikh M., Lombardi L.H., Gattey D.M., Armour R.L., Edmunds B. (2014). Optical Coherence Tomography Angiography of Optic Disc Perfusion in Glaucoma. Ophthalmology.

[31] Mariampillai A., Standish B.A., Moriyama E.H., Khurana M., Munce N.R., Leung M.K.K., Jiang J., Cable A., Wilson B.C., Vitkin A. (2008). Speckle variance detection of microvasculature using swept-source optical coherence tomography. Opt. Lett..

[32] Mahmud M.S., Cadotte D.W., Vuong B., Sun C., Luk T.W.H., Mariampillai A., Yang V. (2013). Review of speckle and phase variance optical coherence tomography to visualize microvascular networks. J. Biomed. Opt..

[33] Braaf B., Donner S., Nam A.S., Bouma B.E., Vakoc B.J. (2018). Complex differential variance angiography with noise-bias correction for optical coherence tomography of the retina. Biomed. Opt. Express.

[34] Zhang A., Zhang Q., Chen C.-L., Wang R. (2015). Methods and algorithms for optical coherence tomography-based angiography: A review and comparison. J. Biomed. Opt..

[35] Reif R., Qin J., An L., Zhi Z., Dziennis S., Wang R. (2012). Quantifying Optical Microangiography Images Obtained from a Spectral Domain Optical Coherence Tomography System. Int. J. Biomed. Imaging.

[36] Li M., Idoughi R., Choudhury B., Heidrich W. (2017). Statistical model for OCT image denoising. Biomed. Opt. Express.

[37] Luisi J., Kraft E.R., Giannos S.A., Patel K., Schmitz-Brown M.E., Reffatto V., Merkley K.H., Gupta P.K. (2021). Longitudinal Assessment of Alkali Injury on Mouse Cornea Using Anterior Segment Optical Coherence Tomography. Transl. Vis. Sci. Technol..

[38] Pi S., Camino A., Wei X., Simonett J., Cepurna W., Huang D., Morrison J.C., Jia Y. (2018). Rodent retinal circulation organization and oxygen metabolism revealed by visible-light optical coherence tomography. Biomed. Opt. Express.

[39] Rocholz R., Teussink M.M., Dolz-Marco R., Holzhey C., Dechent J.F., Tafreshi A., Schulz S. (2018). SPECTRALIS Optical Coherence Tomography Angiography (OCTA): Principles and Clinical Applications. Heidelb. Eng. Acad..

[40] Aguirre-Ramos H., Avina-Cervantes J.G., Cruz-Aceves I., Ruiz-Pinales J., Ledesma S. (2018). Blood vessel segmentation in retinal fundus images using Gabor filters, fractional derivatives, and Expectation Maximization. Appl. Math. Comput..

[41] Rapolu M., Niedzwiedziuk P., Borycki D., Wnuk P., Wojtkowski M. (2018). Enhancing microvasculature maps for Optical Coherence Tomography Angiography (OCT-A). Photon-Lett. Pol..

[42] Gardner M.R., Katta N., Rahman A.S., Rylander H., Milner T.E. (2018). Design Considerations for Murine Retinal Imaging Using Scattering Angle Resolved Optical Coherence Tomography. Appl. Sci..

[43] Ploner S.B., Moult E.M., Choi W., Waheed N.K., Lee B., Novais E.A., Cole E.D., Potsaid B., Husvogt L., Schottenhamml J. (2016). Toward quantitative optical coherence tomography angiography. Retina.

[44] Alam M., Lim J.I., Toslak D., Yao X. (2019). Differential Artery–Vein Analysis Improves the Performance of OCTA Staging of Sickle Cell Retinopathy. Transl. Vis. Sci. Technol..

[45] Schottenhamml J., Moult E.M., Ploner S., Lee B., Novais E.A., Cole E., Dang S., Lu C.D., Husvogt L., Waheed N.K. (2016). An automatic, intercapillary area-based algorithm for quantifying diabetes-related capillary dropout using optical coherence tomography angiography. Retina.

[46] Lin J., Luisi J., Kraft E.R., Giannos S.A., Schmitz-Brown M.E., Gupta P., Motamedi M. (2021). Single-frame optical coherence tomography angiography for the quantification of corneal neovascularization in a mouse model. Ophthalmic Technologies XXXI.

